# Specialization in plant–pollinator networks: insights from local-scale interactions in Glenbow Ranch Provincial Park in Alberta, Canada

**DOI:** 10.1186/s12898-019-0250-z

**Published:** 2019-09-06

**Authors:** Soraya Villalobos, José Manuel Sevenello-Montagner, Jana C. Vamosi

**Affiliations:** 1 Department of Biological Sciences, University of Calgary, Calgary, AB Canada; 2 Department of Biology, University of Ottawa, Ottawa, ON Canada

**Keywords:** Plant–pollinator interaction, Network ecology, Specialization

## Abstract

**Background:**

The occurrence and frequency of plant–pollinator interactions are acknowledged to be a function of multiple factors, including the spatio-temporal distribution of species. The study of pollination specialization by examining network properties and more recently incorporating predictors of pairwise interactions is emerging as a useful framework, yet integrated datasets combining network structure, habitat disturbance, and phylogenetic information are still scarce.

**Results:**

We found that plant–pollinator interactions in a grassland ecosystem in the foothills of the Rocky Mountains are not randomly distributed and that high levels of reciprocal specialization are generated by biological constraints, such as floral symmetry, pollinator size and pollinator sociality, because these traits lead to morphological or phenological mismatching between interacting species. We also detected that landscape degradation was associated with differences in the network topology, but the interaction webs still maintained a consistently higher number of reciprocal specialization cases than expected. Evidence for the reciprocal evolutionary dependence in visitors (e.g., related pollinators visiting related plants) were weak in this study system, however we identified key species joining clustered units.

**Conclusions:**

Our results indicate that the conserved links with keystone species may provide the foundation for generating local reciprocal specialization. From the general topology of the networks, plant–pollinators interactions in sites with disturbance consisted of generalized nodes connecting modules (i.e., hub and numerous connectors). Vice versa, interactions in less disturbed sites consisted of more specialized and symmetrical connections.

## Background

Ecological specialization is usually defined as the tendency of organisms to occupy a restricted niche breadth [1]. This concept has been outlined as a key idea to predict the adaptive response of populations in heterogeneous or fluctuating environments [2]. For example, ecological specialization of flowers, usually referred to as “specialized pollination” [3], is thought have played an important role in angiosperm diversification due to selection by certain pollinators [4]. In this sense, biological constraints forced by phenotypic mismatches in biological attributes (e.g., morphology, phenology and phylogeny) can determine the quantity and quality of species interactions [5, 6]. Floral zygomorphy and pollinator size are good examples of morphological accessibility restrictions that could select for certain pollinator types to visit certain flowers and successfully pollinate them [7]. The specialized pollinator systems of zygomorphic flowers may promote cross-pollination through increased precision in successful pollen deposition on the pollinator’s body. Therefore, bilateral flowers and constant flower-visitors may determine selection for specialization via reproductive isolation between incipient species [7].

Disturbance can have disproportionate effects on specialists within communities either through the effects on abundance of a rare species or due to underlying associations between specialization and traits [8]. The loss of more specialized species and their ecological interactions due to their highly vulnerability to habitat loss are increasingly documented [9, 10]. However, the relative contribution of ecological parameters (e.g., abundance) versus evolutionary parameters (e.g., traits that act as barriers to certain interactions) is often unknown. Currently, the scientific community lacks sufficient information about the variation of most species throughout their ranges from strict specialization to wide generalization as well as which environmental factors underlie these patterns [11–13]. Specifically, within Canadian ecozones, many questions regarding the major effects of disturbance on the loss of species interactions remain unanswered.

Our ability to predict the consequences of species decline depends on our understanding of the evolutionary and ecological consequences of their species interactions [14]. The occurrence and frequency of interactions exhibited by species are quantifiable and depend in part on the spatio-temporal distribution of species [6, 15, 16]. To some extent species may be replaced within communities by close relatives and therefore phylogenetic relationships may provide a framework to examine what determines specific species interactions [17]. The degree to which related members of a community share morphological, life history and ecological characteristics (i.e., phylogenetic signal [18]) can regulate the tendency of closely related species to interact with similar partners or even persist at similar abundance within ecological communities [19]. In this sense, a measure of phylogenetic signal can provide a means of quantifying the degree to which phylogeny influences the structure of interaction webs [20]. Specifically for mutualistic networks, including plant-pollination systems, previous studies have found that animals tend to have more conservatism in their interactions than plants [21], although overall the phylogenetic structure of networks is determined by both animals and plants as a whole [22, 23].

The backbone of ecological networks may rely on the functional role of key species connecting tight clusters of species where species tend to have more specialized interaction. However, the consequences of the loss of these key functional roles are still unclear. Simulation studies have shown conflicting results on the effect of keystone species removal from the web. Some studies have found that networks can be resilient to the loss of some functional roles, but others studies point out the loss of network robustness after the extirpation of specialist species (i.e., connectors) and generalists species (i.e., hubs) [24, 25]. With disturbance, the loss of biodiversity is often accompanied by the erosion of interspecific interactions and the disruption of phylogenetic structure in pollination mutualistic networks [26] but there has been little exploration of whether disturbance disproportionately affects hubs or connectors. The consequences of changes in species composition has been observed to vary with the intensity of land management, as well as features related to the evolutionary history of species [27]. Despite the increasing interest in studying the influence of ecological factors such species richness and species traits in network structure [22, 28]; little is known about the effect of habitat disturbance on the network structure or on the ecologically specialized pairwise interactions (but see [29, 30]). Herein, we used network approaches to obtain estimates of specialization within plant–pollinator networks in Alberta, Canada. Specifically, we examined the cases of reciprocal and asymmetrical species specialization in networks influenced by some degree of landscape transformation (disturbed vs. undisturbed sites). We also evaluated the role of species identity within the network and the influence of phylogenetic signal in species persistence. Finally, we examined the use of nestedness and modularity metrics for the assessment of the sustainability of ecosystems in terms of plant–pollinator interactions.

## Results

### Network descriptors

At study sites at Glenbow Ranch Provincial Park, we observed a total of 42 plant species in flower belonging to 22 families (Additional file 1: Appendix S1). Undisturbed sites hosted 23 flowering plant species and disturbed sites hosted 19 flowering plant species. We validated the differences in landscape disturbance in the study site looking into the historical degradation of the sites. Disturbance regimes in Glenbow Provincial Park affected the taxonomic composition of pollinators as well (Additional file 2: Figure S1). However, despite differences in species identity, the phylogenetic composition was not significantly different for both plants and pollinators between the two sites (Adonis analysis: F_1_, 1.162; P = 0.3 and F_1_ 1.7197; P = 0.2, for plants and pollinators respectively). Surprisingly, we did not find spatial autocorrelation between sites in plant and pollinator composition (Monte-Carlo Mantel test; observation: 0.561; P-value = 0.06; Mantel test; observation: − 0.212; P-value = 0.7).

**Fig. 1:**
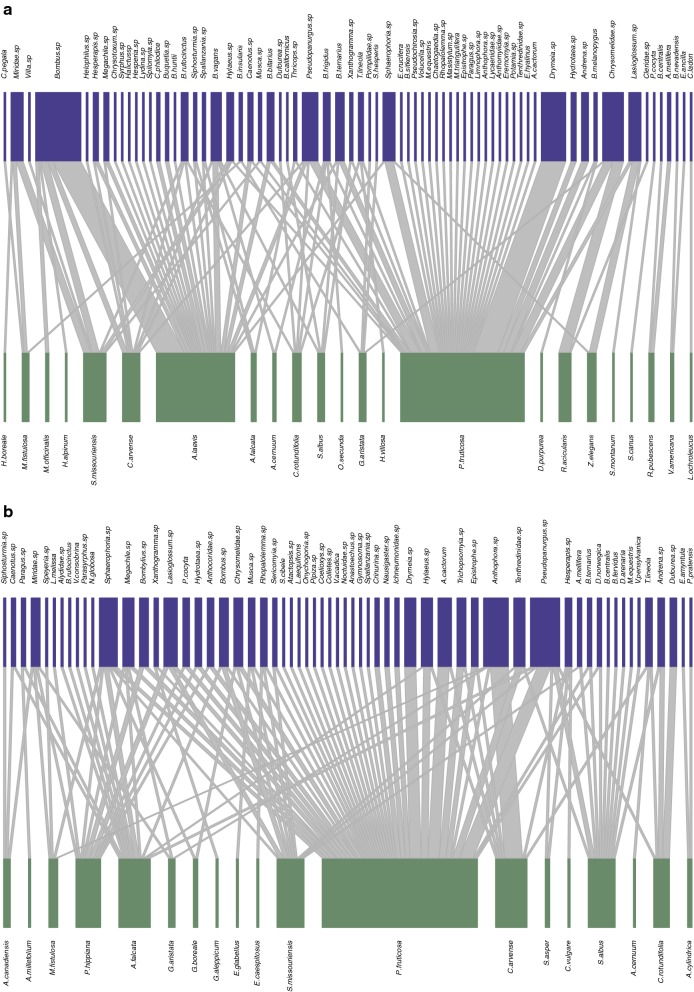
**a** Plant**–**pollinator network for the entire season in undisturbed areas. **b** Plant**–**pollinator network for the entire season in disturbed areas

In the undisturbed sites, we recorded 188 visits throughout the entire season between 70 pollinator species and 23 plant species, resulting in a total of 93 interacting species and a network size of 1610 possible links with an average total of 7.7 interactions per day (Fig. 1a). In general, 12 pollinators could be considered to have a high specialization index (d′ > 0.7, Table 1A), with the Lepidoptera *Phyciodes cocyta* and *Celastrina ladon* showing the greatest specialization level. Conversely, the least specialized pollinators were hoverflies *Parasyrphus* spp., *Merodon* and *Paragus* (Additional file 1: Appendix S1). The most specialized plant species were members of Fabaceae (*Lathyrus ochroleucus*, *Vicia americana*) and Asteraceae (*Senecio canus*; Table 1B).

**Table 1:** (A) Species-level descriptors for the more specialized pollinators (d′ > 0.7) calculated for 54 quantitative plant–pollinator networks; (B) species-level descriptors for the more specialized plants (d′ > 0.7) calculated for six quantitative plant–pollinator networks

	Species	Family	d**′**	Ratio (visits/species)
Panel (A)				
Undisturbed	*Bombus centralis*	Apidae	0.7	2:2
	*Bombus nevadensis*	Apidae	0.8	1:1
	*Euphilotes ancilla*	Lycaenidae	0.8	1:1
	*Phyciodes cocyta*	Nymphalidae	1.0	1:1
	*Celastrina ladon*	Lycaenidae	1.0	1:1
Disturbed	*Dufourea* sp.	Halictidae	0.7	3:1
	*Siphosturmia* sp.	Tachinidae	0.7	1:1
	*Everes amyntula*	Lycaenidae	0.8	2:1
	*Phyciodes pratensis*	Nymphalidae	0.8	3:2
Panel (B)				
Undisturbed	*Aster falcata*	Asteraceae	0.75	3:3
	*Dalea purpurea*	Fabaceae	0.78	1:1
	*Senecio canus*	Asteraceae	1	1:1
	*Lathyrus ochroleucus*	Fabaceae	1	1:1
	*Rubus pubescens*	Rosaceae	1	3:2
	*Vicia americana*	Fabaceae	1	2:2
Disturbed	*Anemone canadensis*	Ranunculaceae	0.7	4:3
	*Achillea millefolium*	Asteraceae	0.7	1:1
	*Geum aleppicum*	Rosaceae	0.7	1:1
	*Anemone cylindrica*	Ranunculaceae	1.0	2:2

Proportion and ratio indicate the number of plant species visited per day

In the disturbed sites, we recorded 155 visits between 61 pollinator species and 19 plant species (Fig. 1b). This resulted in a total of 80 interacting species and a network size of 1159 possible links with an average total of 9.2 interactions per day (Fig. 1b). Six pollinator species showed high specialization index (d′ > 0.7, Table 1A), and the Lepidoptera *Phyciodes pratensis* and *Everes amyntula* were again the most specialized pollinator species (Table 1A). The least specialized pollinators were *Bombus* sp., and the syrphids *Epistrophes* sp. *and Pipiza* spp. (Additional file 1: Appendix S1). The plant species with the highest specialization index was *Anemone cylindrica* (Ranunculaceae) (Table 1B).

In general, the explanatory power of pollinator size and the degree of sociality (eusocial-solitary), determined the specialization of the pollinators and symmetry determined plant specialization. Based on AIC values, the flower symmetry model showed the best fit to the specialization level from the plants side (Table 2) yet all three models had similarly low AIC values (∆AIC < 2). For pollinators, however, the evidence ratio showed that sociality has 86% of normalized probability to be the best model than the pollinator size model in terms of Kullback–Leibler discrepancy.

**Table 2:** Summary of terms for generalized mixed-effects models (GAMMs) on the effects of body size and sociality on specialization of pollinator species (d′) and the effect of flower abundance and symmetry on specialization of plant species

Model	AIC_c_	*W*
d′poll ~ size + sociality	40.331	0.7
d′poll ~ size	37.425	0.03
d′poll ~ sociality	35.203	0.2
d′plant ~ flower abundance + symmetry	6.7700	0.5
d′plant ~ flower abundance	6.2944	0.3
d′plant ~ symmetry	5.2212	0.05

Study site was included as random effect

*AIC*_*c*_ Akaike information criterion*, w*_*i*_ Akaike weights

**Fig. 2:**
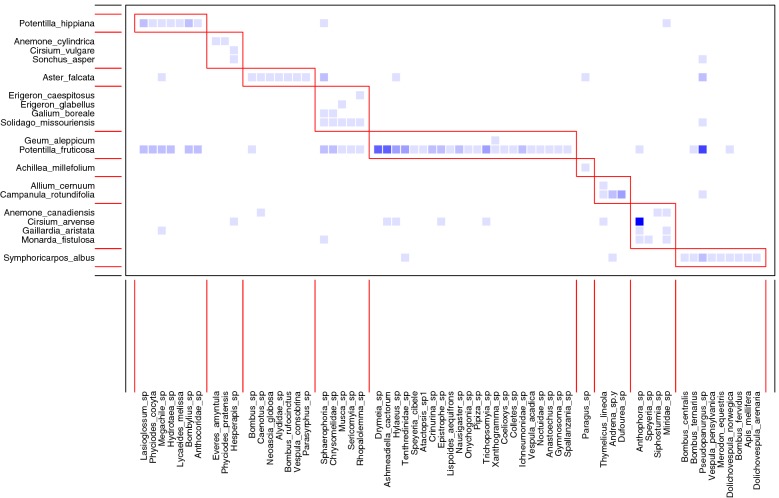
Interaction network in the disturbed site showing module organization. Plants at the left and pollinators at the bottom. The color intensity indicates the interaction frequency between partners. The subsets in red boxes features modules calculated using computeModules functions in bipartite [25]

**Fig. 3:**
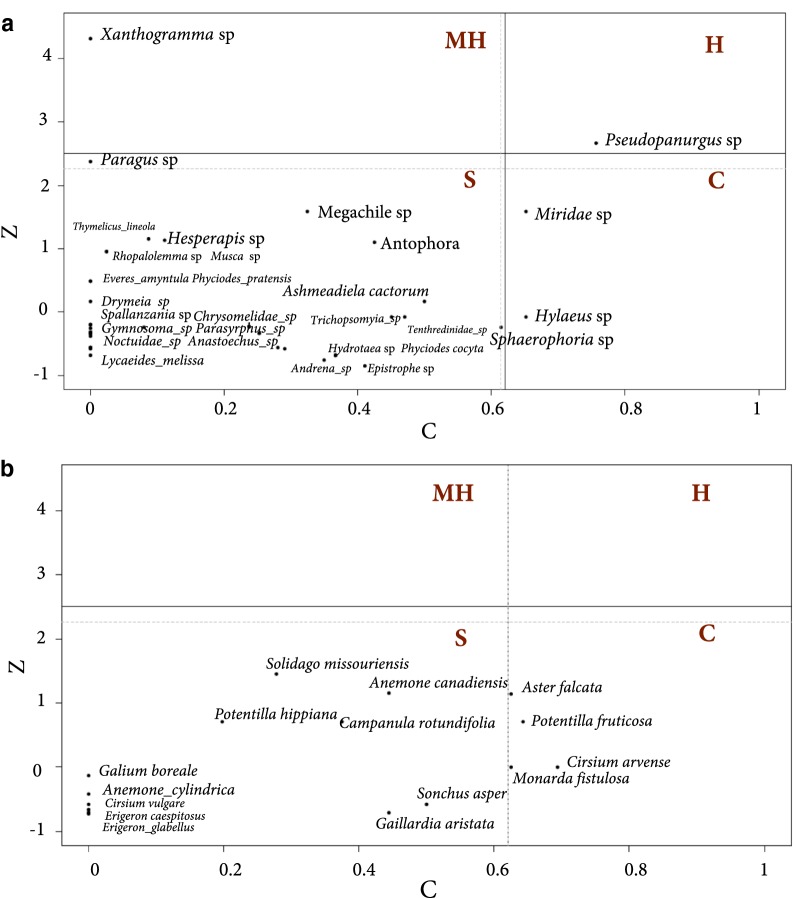
Distribution of plant pollinator interaction according to their network role [15] for **a** pollinators and **b** plants in disturbed sites. *S* specialists or peripheral, *C* connectors, *H* hub and MH Module Hub. Black lines indicate critical values according to Olesen et al. [15], gray line indicate 95% quantiles from 1000 null models

Networks in both disturbed and undisturbed sites were significantly more specialized than expected (Table 3). However, only the networks calculated from undisturbed sites were significantly more nested than expected (Table 3). The observed modularity Q revealed that both networks were significantly modular compared to the null model (Table 3, Fig. 2). In disturbed sites, the among-module connectivity (c) values ranged from 0 to 1 with two species of pollinators (*Hylaeus* spp. and *Miridae* spp.) and two species of plants (*Aster falcata* and *Monarda fistulosa*) exceeding the threshold of 0.625. The within-module degree (z) values ranged between − 0.5 and 3 with no plant species but four pollinator species (*Ashmeadiella cactorum*, *Drymeia* spp., *Paragus* spp. and *Pseudopanurgus* spp.) exceeding the value of 2.5 (Fig. 3). However only one species, *Pseudopanurgus* spp. (Andrenidae) constituted a hub species, exceeding both thresholds (Fig. 3). The majority of non-hub species from both guilds were peripheral, except for the connectors *Hylaeus* sp. and *Miridae* spp. and *Aster falcata*.

**Table 3:** Network level descriptors calculated for disturbed and undisturbed networks

Descriptor	Undisturbed		Disturbed	
	Observed	P	Observed (%MO)	P
Network specialization H2	0.5 (0.7%)^a^	0.000	0.35(0.8%)^a^	0.001
Nestedness NODF	8.8 (32%)^a^	0.000	12 (40%)^a^	0.46
Modularity (Q)	0.5 (11.48)^b^	0.001	0.4 (3.9)^b^	0.001

^a^Percentage of network descriptor value relative to the mean of the descriptor for 1000 randomly generated networks of the same size determined with r2dtable algorithm

^b^z-scores for Modularity Q (computed using computeModules functions in bipartite)

We found phylogenetic signal in the abundance of pollinators only for the disturbed sites (Table 4), indicating phylogenetic dependence in the prevalence of species in this area. The overall strength of the phylogenetic signal for the linear model fitted to each network in both disturbed and undisturbed sites (MSE_data_, Table 5) was much closer to the values found in the assumption of no phylogenetic signal or star phylogeny (MSE_star_), than for the assumption of maximum phylogenetic signal or Brownian model (MSE_brownian_). Thus, these results indicate that in disturbed sites, closely related pollinators interacted with similar suites of plant species (but the same was not true for closely-related plants) and contributed to the phylogenetic structure of the plant–pollinator network, yet there was a weak phylogenetic signal in the interaction patterns. Conversely, within the undisturbed sites, the phylogenetic relationships of both plants and pollinators contributed to the network structure, but similar to the disturbed sites, the overall phylogenetic signal for reciprocal interaction was weak.

**Table 4:** Phylogenetic signal in plant and pollinator species persistence based in their abundance

Guild	Site	K	P-value	Conclusion
Pollinator	Undisturbed	0.171	P = 0.203	No Signal
	Disturbed	0.309	P = 0.05	Signal
Plants	Undisturbed	0.052	P = 0.515	No Signal
	Disturbed	0.023	P = 0.473	No Signal

P-value is the test for non-random signal

**Table 5:** The phylogenetic signal on the strength of plant–pollinator interaction for six networks in the Rocky Mountains

Site ID*	*d*_*plant *_	*d*_*poll *_	MSE_Data_	MSE_Star_	MSE _Brownian_
Site 1	2.8^−05^ (2.2^−07^–0.099)	0.01 (8.12^−27^–0.32)	0.49	0.52	0.99
Site 2	8.5^−11^ (7.26^−11^–0.169)	0.36 (0.13–0.60)	0.20	0.25	0.40
Site 3	4.02^−12^ (2.4^−10^–0.017)	0.68 (0.54–0.78)	0.30	0.38	0.61
Site 4	1.69^−04^ (1.17^−08^–0.193)	2.14^−12^ (2.70^−14^–0.001)	0.14	0.14	0.34
Site 5	2.40^−28^ (0.00–8.28^−10^)	0.85 (0.37–1.04)	0.44	0.62	0.33
Site 6	2.05^−11^ (0–2.35^−29^)	0.54 (0.34–0.66)	0.28	0.25	15.1

d_plant_ and d_poll_ intervals measures the strength from plant phylogeny and pollinators, respectively. Measures include confidence intervals (CI)

*Sites 1–3 correspond to undisturbed areas. Sites 4–6 correspond to disturbed sites

The *nodesig* analysis (see “Methods”) indicated that the clades represented at greater frequency than expected from the null model were *Andrena* and *Pseudopanurgus* in disturbed sites (Table 6) and Apini and *Bombus* in undisturbed sites. The less represented clades were Syrphidae and Tachinidae in disturbed areas and Vespidae in undisturbed areas. In plants, overrepresented clades were Asteraceae in the undisturbed sites and *Potentilla* and *Rubus* in the disturbed ones.

**Table 6:** Clades significantly contributing to the plant–pollinator network structure in two different sites of foothills Rocky Mountains (Alberta)

Guild	Habitat	More	Less
Pollinators	Disturbed	*Andrena*	Syrphidae
		*Pseudopanurgus*	Tachinidae
	Undisturbed	Apini *Bombus*	Vespidae
Plants	Disturbed	*Potentilla*	
		*Rubus*	
	Undisturbed	Asteraceae	

*Nodesig* algorithm phylocom

## Discussion

The degree to which ecological parameters such as abundance versus morphological parameters (which are heavily influenced by evolutionary history) determine pairwise interactions between trophic levels remains a difficult challenge for evolutionary ecologists [31]. The network properties that we measured support the prediction that plant–pollinator interactions are not randomly distributed but are instead generated by biological constraints forced by morphological mismatches between interacting species as well as abundance differences caused by habitat preferences [32, 33]. Overall, we found that species and network-level specialization were influenced by habitat disturbance, leading to differences in interaction partners and network size, as well as network topology. Likewise, we detected non-random patterns of phylogenetic representation in clades present in the different disturbance sites, however we found only limited evidence of evolutionary history playing a role in visitation (e.g., related pollinators visiting similar plants).

We chose specialization measures that are largely robust against variation in matrix size, shape, and sampling effort (i.e., d′ specialization index). Species with high d′ values were: (1) bilateral flowers from the pea family (*Lathyrus ochroleucus*, *Vicia americana*) that were visited by Lepidoptera (e.g., *Celastrina ladon*, *Euphilotes ancilla*), and (2) tubular-ligulate flowers (*Achillea millefolium*, *Senecio canus*) that were visited by a suite of bees. These results could suggest, in both cases, a symmetrical dependence for the morphological fit between flower and its pollinator [34]. Highly reciprocal plant–pollinator specialization rates may indicate that co-evolutionary processes have led to successful pollen deposition between conspecific flowers [35]. Specifically, the bilateral symmetry seen in *L*. *ochroleucus* and *V*. *americana* may represent targets of special conservation interest to preserve unique interspecific relationships [7]. High values of d′ were also observed in radial flowers (*Anemone cylindrical*, *A. canadensis*, *Rubus pubescens*) mostly visited by beeflies (e.g., *Paragus* sp.) and the honey bee (*Apis mellifera*). For the interaction between radially symmetrical flowers and beeflies, we did not detect increased seed set for the plant species visited by beeflies (Villalobos, unpublished data). Therefore, we infer that the high level of specialization that we detected between those species could be related to the large flowers providing space for bee fly ovipositioning rather than pollinator specialization [28]. However, we further require testing the role of the reproductive biology of these species within this ecosystem.

At the network level, both undisturbed and disturbed networks showed higher specialization (H2′) than expected by chance. This result suggests that despite the landscape degradation in the disturbed site, both networks showed similar structure in terms of trait matching and phenological constraints. This finding is consistent with previous studies where the level of landscape degradation had minimal effects on certain networks properties [36]. Pollinator size was important for pollinator specialization and floral symmetry (and, to a lesser degree, plant abundance) was important for plant specialization. It has been hypothesized that solitary bees might be more specialized in their visitation rates than social species [37], yet in this study social bees had larger specialization index (d′) than solitary pollinator species. Interestingly, although that may suggest that social bees may be more selective in their visits, we found some bees with a high specialization index that have been previously reported as highly generalist (e.g., *Bombus nevadensis*; [38]). These differences may arise because the d′ index takes into account the abundance of species in the community. For example, a pollinator species generates the minimum specialization index when it is observed visiting the most dominant plant species even if it visits only one plant species [39]. In our study system, most of species with high specialization index registered 1:1 daily visit ratio (i.e., number of pollinator visits per day Table 1A, B). Specifically, *Bombus nevadensis* visited only *Vicia americana*. That plant species also showed a high specialization index with a low abundance record, which is consistent with high reciprocal interaction based on the available resources within the community. Hence, future studies could clarify the effect of sample size and variance on the detection of differences between pairwise comparison for symmetry and pollinator size [40].

Networks in more undisturbed areas were significantly less nested than expected (*P *< 0.05). This output supports the high levels of specialization (i.e., densely clustered network) identified with H2 and d′ in the undisturbed sites, indicating that (1) plant–pollinator interactions are more specialized than previously thought, at least in prairie grasslands and/or; (2) that pollinators prefer to exploit plants that are not being visited by many other species. Our result departs from those previous studies where the association in mutualistic plant–pollinator systems has been described as being nested and highly generalized compared to plant–herbivore or host-parasite webs [5, 41, 42]. Previous studies have pointed out that some parameters of the network structure such as nestedness, depends on how the underlying data is collected and analyzed [43, 44]. For example, the model of nestedness in natural communities compared to simulated communities has more explanatory power when interaction-specific species traits (i.e., forbidden links) is introduced in the analysis [45]. Here, we attempted to maximize the quantitative importance of the links using weighted data, species traits and metrics based on weighted links.

### The role of species within modular networks

Modules are subsets of networks formed by species that tend to interact more between each other than with species from other modules [46]. We identified only modular organization within the disturbed area. This compartmentalization of plant–pollinator communities may also be consistent with the idea that modular interactions tend to emerge in networks where there is spatial or temporal segregation of interacting species, which may occur in disturbed areas [47]. Interestingly, we observed that many pollinators from several orders were present in all modules, however only one visitor species *Pseudopanurgus* sp. (Andrenidae) represented a hub species (i.e., generalist species). Some species of this genus are specialists on Asteraceae (e.g., *Pseudopanurgus compositarum* [19]), which could explain the connector roles (i.e., specialist species) found in the plant modules where Asteraceae species were abundant (e.g., *Aster falcata*). Likewise, we detected connector topological roles for the non-hub pollinator species supporting the modules to which plants are connected.

Modules may reflect units bounded by evolutionary constraints such as niche segregation (e.g., differences in floral traits) [5]. We also detected phylogenetic signal in pollinator abundance; therefore, phylogenetic relatedness is a factor determining interaction patterns that may drive clustered links with more potential for generating local reciprocal specialization [24]. Hub and connector species could be considered as network keystone species [31]. In this study, these species were *Pseudopanurgus* spp. and *Hylaeus* spp. (pollinator species) and *Aster falcata*, *Potentilla fruticosa*, *Monarda fistulosa* and *Cirsium arvense* (plant species). If these species represent the backbone on which the network is built, they can be considered critical for maintaining the overall network structure in the most disturbed areas of Glenbow Ranch Provincial Park [48]. It is currently unclear how the network stability would operate with the loss of a hub species. To better understand the consequences of species loss, future studies should examine other factors such as the quality effect of pollinator visitation upon plant partners (e.g., seed production).

### Evolutionary implications

The inheritance of morphological, life history and ecological characteristics from common ancestors (i.e., seen as phylogenetic signal, [49]) can govern the tendency of closely related species to interact with similar partners [50]. We detected non-random patterns of phylogenetic representation in clades present in both areas. However, phylogenetic signal in species interactions as well as the prevalence of species was only detected for pollinators in the disturbed assemblages. Overrepresented clades in disturbed sites (e.g., *Andrena* and *Pseudopanurgus*) may indicate that these groups are being filtered by certain features of the disturbed environment (e.g., environmental characteristics and soil types that favour nest constructions [51]). The disturbed site was located in open bare soil areas influenced by high rates of evapotranspiration, which could facilitate nest construction. Species from the genera *Andrena* and *Pseudopanurgus* construct underground and multicellular nests in habitats with good water irrigation [36]. Finally, we did not find strong evidence of the reciprocal evolutionary dependence in visitors (e.g., related pollinators visiting similar species of plants). However, with the strong phylogenetic signal detected in pollinators but not in plants, we infer that pollinators tend to have more conservatism in their interactions than do plants [22, 30]. Thus, consistent with previous studies [52], there does seem to be a weak influence of phylogeny on network structure and specific species interactions.

## Conclusion

This study detected cases of reciprocal specialization that may deserve particular attention for conservation. Landscape degradation led to differences in the network topology, but interactions still maintained a consistent number of reciprocal specialization cases than expected under neutral conditions. From the general topology of the networks, plant–pollinators interactions in sites with a long history of disturbance consisted of generalized nodes connecting modules (i.e., hub and numerous connectors). Conversely, interactions in less disturbed areas were based on tighter and symmetrical connection between specialists. The implications for network functioning reflected on the topological role of hub and connector’s species within disturbed sites, and the reciprocal specialization detected in less-impacted sites may indicate conservative units bounded by evolutionary constraints. We anticipate that conservative links in keystone species may represent potential for generating local reciprocal specialization.

## Methods

### Visitation data

Plant–pollinator visitation data were collected at six sites in Glenbow Ranch Provincial Park. The park is located along the north shore of the Bow River between Calgary and Cochrane (AB) and consists of 1300 hectares of foothills (Glenbow Ranch Foundation http://www.grpf.ca/). The area contains fescue grassland, prairie and plateaus with deciduous and mixedwood forests at lower elevations. The land use history of the area has incorporated a number of different activities, such as farming and livestock grazing. The study sites were chosen by taking into account the management level (i.e., highly fragmented vs. non-fragmented habitats). The most fragmented sites corresponded to areas intensively worked as ranch land until 2008. These areas exhibit high levels of soil erosion and soil compaction due to the grazing activities for more than 20 years (Glenbow Ranch Foundation http://www.grpf.ca). The most preserved areas corresponded to deciduous and mixedwood forests at lower elevations with a low record of degradation. Aspen poplar *Populus tremuloides* and white birch *Betula papyrifera* dominate the tree layer section. However, they occurred in low density in the study plots.

All six sites were separated from the others by 0.5 km. At each site, we set-up three parallel 25 m transects. Transects were aligned in an east–west direction and no closer than 50 m from the edge of the field. All flower resources were located at the herbaceous level. Insect pollinated shrubs and small trees were sparse and not included. Visitation rate was surveyed from June to August 2015. All sites were sampled eight times during mid-season with a minimum of 5 days between sampling. All insects were taxonomically identified and deposited in the museum collection in the department of biological sciences at the university of Calgary.

### Aerial net sampling

Walking slowly within transects, we collected insects observed visiting flowers up to 1 m either side of the transect line and 1 m ahead using an aerial net, with a sampling period of 5 min/line. One minute was added for each insect capture for processing (handling, placing in jar and labelling) for an estimated 20–40 min/transect. Bee surveys were conducted late morning and early afternoon at each site by walking approximately 0.3 m/s (~ 5 min per transect). All floral visitors were placed in individual vials with information on species of flower visited, transect number and section, date and time.

### Floral census plots

Floral abundance was estimated by counting the number of open floral units, in five 1-m^2^ plots spaced at 5 m intervals along each transect. In addition, flower counts were made at each site during each sample day. All open, bee visited flowers were identified and counted within 1 m on either side of each transect, and summed for each 25 m increment (e.g., 0–25 m, 25–50 m, etc.) [6].

### Network descriptors

From the observed flower-visitor interaction data, we created a quantitative visitation network matrix for each site. The total species richness or network size (SR) was calculated as the sum of the total number of pollinator species and plant species per network. We used flower visitation as a proxy for the potential for pollination, taking into account that visitation rate is often positively correlated with fruit set [42]. We quantified the total number of interacting species and estimated the total network size (i.e., the total possible links) multiplying the number of total pollinators by the total number of plants [34].

To measure the specialization level of species, we used the species specialization index (d′) [53], which measures the reciprocal specialization index of the species within the network. d′ is highly robust to sample size, and ranges from 0 (no specialization) to 1 (perfect specialists). The d′ index takes into account the abundance of species in the community. For example, a pollinator species generates the minimum specialization index when it is observed visiting the most dominant species even if it visits only one plant species [53]. In contrast, it generates the maximum specialization value when it visits only the two rarest plant species [54].

Calculations were made on single networks per site generated from the combination of the three sub-networks sampled in each site. We used a generalized mixed-effects model (GLMM) to assess the effects of zygomorphy, flower abundance, pollinator sociality (i.e., Hymenoptera) and body size on specialization level of plants and pollinators (d′) (Additional file 1: Appendix S1). All the quantitative variables exhibited normal error distributions (i.e., pollinator size and flower abundance). We examined the effect of pollinators in the model as d′ ~ pollinator size + pollinator sociality, and the effects of plant traits with the model d′ ~ symmetry + colour + flower abundance. In both models, study site (i.e., disturbed and undisturbed) was included as a random effect. To choose the model(s) that best predicts specialization level, we calculated Akaike’s information criterion for each model (AIC = 2l_i_ + 2k, where l_i_ is the log-likelihood of model *i* and *k* is the number of parameters estimated from the data). AIC is an estimator of the relative quality of statistical models (i.e., it provides a mean for model selection, [54]). To compare the relative fit of competing models, we calculated AIC_c_, a second-order AIC, necessary for small samples n/k < 40 [55]. In addition, we used Akaike’s weight (w) for the closets candidate models [54], using the equation:

1 $$w = \frac{{e^{{ - 2\Delta \left( {AIC} \right)}} }}{{\mathop \sum \nolimits_{k = 1}^{K} e^{{ - 2\Delta \left( {AIC} \right)}} }}.$$

Finally, we calculated the normalized evidence ratio for all models:

2 $$\frac{{ w_{Ai} \left( {AIC} \right)}}{{w_{Aj} \left( {AIC} \right) + w_{Ai} \left( {AIC} \right) }}$$

where, $w_{Ai} \left( {AIC} \right)$ shows the set of Akaike weights for the illustrative data.

Akaike’s weight is interpreted as the probability that a given model is the better model that minimizes the Kullback–Leibler discrepancy (i.e., minimize the divergence of probability distribution; [56]).

At the network level, we inferred specialization with H_2_, which measures the overall level of dependence of each interacting species on its partners [57], as well as a measured of weighted nestedness (weighted NODF2 metric [53]). To reduce the effect caused by the network size over the number of links between interacting species, we standardized these parameters using Z scores against 1000 random network models using ‘r2dtable’ method implemented in Vegan R [53]. The r2dtable function yields a null model that preserves the original number of links, thus connectance is the same as in the original network. We also calculated modularity (Q) using NetworkLevel and computeModules functions implemented in bipartite [53]. The absolute value of modularity (Q) was compared to a null model standardized to *Z* scores [58]:

3 $${\text{ZQ}} = \frac{{{\text{Qobserved}} - {\bar{\text{Q}}\text{null}}}}{{\upsigma{\text{null}}}}$$

Since z-scores are assumed to be normally distributed, values of ZQ above 2 are considered significantly modular.

To identify the roles of species within the networks, for each species we identified the module arrangement using computed standardised among-modules connectivity (C) and within-module degree values (Z; a metric of how well connected a given species is to other species within the same module) [58]. We computed these indices on the number of links per species, weighted by the number of interactions per link. Critical values of c and z are 0.625 and 2.5, respectively [58]. Species exceeding both of these values are called “hubs” at network level because they link different partners at different locations and times [24]. Species were also classified as: specialists or peripheral (low Z and low C), specialist connectors (low Z and high C), Module Hub (high Z and low C), and hubs (high Z and high C) [59].

### Phylogenetic signal in plant*–*pollinator persistence

We generated a regional plant phylogeny using the online software Phylomatic [60] for all plants as well as all pollinator species in the dataset. To time-calibrate the branch lengths on the plant phylogeny, we applied ‘Wikstrom’ ages to internal nodes [59] with the BLADJ algorithm. This algorithm provides approximate branch lengths, so that aging a tree node is at least as old as the age given [33].

For pollinators, we used an insect supertree with previously adjusted branch lengths, sensu Chamberlain et al. [61]. To represent uncertainty in the topology [62], the resolution of the supertree was standardized to genus level with polytomies linking species within genera [59].

To determine which particular clades of both plants and pollinators are represented more or less than expected by chance across the networks, we used the Nodesig algorithm in Phylocom [63]. *Nodesig* tests each node for overabundance of terminal taxa distal to it and locates the position of phylogenetic clustering or overdispersion. In other words, *Nodesig* identifies clades responsible for phylogenetic structure [64]. In this analysis, we employed a randomization model that maintains species richness in each sample but randomizes the identity of species.

To measure the phylogenetic signal in species community abundance (i.e., species prevalence within communities), we calculated the K statistic [65]. K is a measure of phylogenetic signal that compares the observed signal in a given trait to the signal under a Brownian motion model (i.e., where trait evolution follows a random walk along the branches of the phylogenetic tree). K values of 1 correspond to a Brownian motion process, where closely-related species are more similar in trait values than expected by random chance but not through selection. K values greater than 1 indicate strong phylogenetic signal and conservatism of traits. If there is a phylogenetic signal in the prevalence of species, phylogenetic structure among communities (and thus, in interactions) may emerge as a consequence [20].

To quantify whether or not closely related pollinator species are more likely than expected by chance to interact with the same suite of plant species (and vice versa, if the attraction of pollinator species exhibits a phylogenetic signal in plants), we used Mantel tests to compare covariance matrix for phylogenetic distance with matrices of ecological distance [20]. This approach uses a linear model approach, fitting the phylogenetic variance–covariance matrix to the interaction matrix to quantify the effect of phylogeny on species interaction patterns. Therefore, if the visitation of plants by pollinators is driven by traits with strong phylogenetic signals, plants and pollinator pairs should be correlated. The analysis resulted in two independent measures of the phylogenetic signal (d), one for plants and another for pollinators, as well as an overall measure of the strength of phylogenetic signal of both phylogenies fitted in a model for the entire community (mean square error, MSE). Values of d = 0 represent a lack of phylogenetic correlation, whereas d = 1 represents a maximum correlation of phylogenetic signals [22]. Overall strength of the phylogenetic signal on the interaction matrix was evaluated in three models one assuming no phylogenetic signal (dplants = dpollinators = 0; Star model i.e., removing the effect of phylogenetic signal), a second model assuming a maximum phylogenetic signal (dplants = dpollinator = 1; Brownian model) and a final model incorporating the observed phylogenetic signals combined (estimated dplants and dpollinators; Data model).

## Supplementary information

**Additional file 1: Appendix S1.** Species traits and abundance data for plants and pollinators in Glenbow Ranch.

**Additional file 2: Figure S1.** Bray-Curtis dissimilarity index of visitor and plant composition of undisturbed (UD) and disturbed (D) sites. The index approaches 0 when samples are similar and approaches 1 when assemblages are different. Visitors composition differ in their clade compositional structure between undisturbed and disturbed sites.

## Data Availability

The datasets used and/or analysed during the current study are available from the corresponding author on reasonable request.

## Abbreviations

MSE: mean square error
d′: specialization index of species
d: phylogenetic signal
H2′: the total specialization index at the entire network
SR: the total species richness or network size
GLMM: generalized mixed-effects model
AIC: Akaike’s information criterion
Q: modularity
C: standardised among-modules connectivity
Z: metric of within-module connectance
Nodesig: tests for overabundance of terminal taxa

## References

[1] Elton C (1927). Animal ecology. Nature.

[2] Levins R (1968). Evolution in changing environments : some theoretical explorations.

[3] Armbruster WS (2017). The specialization continuum in pollination systems: diversity of concepts and implications for ecology, evolution and conservation. Funct Ecol.

[4] Darwin C (1876). The effects of cross and self fertilisation in the vegetable kingdom.

[5] Bascompte J, Jordano P, Melia CJ (2003). The nested assembly of plant—animal mutualistic networks. Proc Natl Acad Sci.

[6] Vázquez DP, Blüthgen N, Cagnolo L, Chacoff NP (2009). Uniting pattern and process in plant-animal mutualistic networks: a review. Ann Bot.

[7] Sargent RD (2004). Floral symmetry affects speciation rates in angiosperms. Proc Biol Sci.

[8] Dulvy NK, Fowler SL, Musick JA, Cavanagh RD, Kyne M, Harrison LR (2014). Extinction risk and conservation of the world’s sharks and rays. eLife.

[9] Devictor V, Clavel J, Julliard R (2010). Defining and measuring ecological specialization. J Appl Ecol.

[10] Weiner CN, Werner M, Linsenmair KE, Blüthgen N (2011). Land use intensity in grasslands: changes in biodiversity, species composition and specialisation in flower visitor networks. Basic Appl Ecol.

[11] Sheffield CS, Museum RS, Street A. The Bees (Hymenoptera : Apoidea, Apiformes) of the prairies ecozone with comparisons to other grasslands of Canada. In: Giberson DJ, Cárcamo HA, editors. Arthropods of Canadian grasslands (volume 4): biodiversity and systematics part 2. Biological Survey of Canada; 2014. p. 427–67.

[12] Adderley LJ, Vamosi JC (2015). Species and phylogenetic heterogeneity in visitation affects reproductive success in an island system. Int J Plant Sci.

[13] Muir JL, Vamosi JC (2015). I Invasive Scotch broom (*Cytisus scoparius*, Fabaceae) and the pollination success of three Garry oak-associated plant species. Biol Invasions.

[14] Memmott J, Waser NM, Price MV (2004). Tolerance of pollination networks to species extinctions. Proc R Soc.

[15] Olesen JM, Bascompte J, Dupont YL, Jordano P (2007). The modularity of pollination networks. PNAS.

[16] Dupont YL, Olesen JM (2012). Stability of modular structure in temporal cumulative plant—flower-visitor networks. Ecol Complex.

[17] Rezende EL, Lavabre JE, Guimarães PR, Jordano P, Bascompte J (2007). Non-random coextinctions in phylogenetically structured mutualistic networks. Nature.

[18] Martins E (2000). Adaptation and the comparative method. Trends Ecol Evol.

[19] Dupont YL, Olesen JM (2009). Ecological modules and roles of species in heathland plant—insect flower visitor networks. J Anim Ecol.

[20] Ives AR, Godfray HCJ (2006). Phylogenetic analysis of trophic associations. Am Nat.

[21] Rohr RP, Bascompte J (2014). Components of phylogenetic signal in antagonistic and mutualistic networks. Am Nat.

[22] González AMM, Dalsgaard B, Nogués-bravo D, Graham CH, Schleuning M, Maruyama PK (2015). The macroecology of phylogenetically structured hummingbird—plant networks. Glob Ecol Biogeogr.

[23] Vazquez DP, Poulin R, Krasnov BR, Shenbrot GI (2005). Species abundance and the distribution of specialization in host–parasite interaction networks. J Anim Ecol.

[24] Goldstein J, Zych M (2016). What if we lose a hub? Experimental testing of pollination network resilience to removal of keystone floral resources. Arthropod Plant Interact.

[25] Montoya M, Pimm SL, Sole RV (2006). Ecological networks and their fragility. Nature.

[26] Aizen MA, Sabatino M, Luis J (2016). The phylogenetic structure of plant—pollinator networks increases with habitat size and isolation. Ecol Lett.

[27] Rollin O, Benelli G, Benvenuti S, Decourtye A, Wratten SD, Canale A (2016). Weed-insect pollinator networks as bio-indicators of ecological sustainability in agriculture. Agron Sustain Dev.

[28] Olito C, Fox JW (2015). Species traits and abundances predict metrics of plant—pollinator network structure, but not pairwise interactions. Oikos.

[29] Vanbergen AJ, Woodcock BA, Heard MS, Chapman DS (2017). Network size, structure and mutualism dependence affect the propensity for plant—pollinator extinction cascades. Br Ecol Soc.

[30] Chamberlain SA, Cartar RV, Worley AC, Semmler SJ, Gielens G, Elwell S (2014). Traits and phylogenetic history contribute to network structure across Canadian plant–pollinator communities. Oecologia.

[31] Traveset A, Tur C, Eguíluz VM (2013). Plant survival and keystone pollinator species in stochastic coextinction models : role of intrinsic dependence on animal-pollination. Proc R Soc.

[32] Bascompte J, Jordano P, Olesen JM (2006). Asymmetric coevolutionary networks facilitate biodiversity maintenance. Science.

[33] Vamosi JC, Moray CM, Garcha NK, Chamberlain SA, Mooers AØ (2014). Pollinators visit related plant species across 29 plant–pollinator networks. Ecol Evol.

[34] Blüthgen N, Menzel F, Blüthgen N (2006). Measuring specialization in species interaction networks. BMC Ecol.

[35] Lázaro A, Totland O (2014). The influence of floral symmetry, dependence on pollinators and pollination generalization on flower size variation. Ann Bot.

[36] Nielsen A, Totland Ø (2014). Structural properties of mutualistic networks withstand habitat degradation while species functional roles might change. Oikos.

[37] Zurbuchen A, Landert L, Klaiber J, Müller A, Hein S, Dorn S (2010). Maximum foraging ranges in solitary bees: only few individuals have the capability to cover long foraging distances. Biol Conserv.

[38] Macior LW (1974). Pollination ecology of the front range of the Colorado Rocky Mountains. Melanderia.

[39] Iriondo J, Strauss R, Vazquez D, Bluethgen N, Clauset A, Strauss R. Package ‘bipartite.’ 2015;1–8.

[40] Mundfrom DJ, Piccone A, Perrett JJ, Schaffer J, Roozeboom M (2006). Bonferroni adjustments in tests for regression coefficients. Mult Linear Regres Viewp.

[41] Bascompte J, Jordano P (2007). Plant–animal mutualistic networks: the architecture of biodiversity. Annu Rev Ecol Evol Syst.

[42] Bascompte J (2009). Mutualistic networks. Front Ecol Environ.

[43] Ballantyne G, Baldock KC, Willmer PG (1814). Pollinator importance networks—visitation and pollen deposition in a heathland plant community. Proc R Soc B.

[44] Krishna A, Guimara PR, Jordano P, Bascompte J (2008). A neutral-niche theory of nestedness in mutualistic networks. Oikos.

[45] Krause AE, Frank KA, Mason DM, Ulanowicz RE, Taylor WW (2003). Compartments revealed in food-web structure. Nature.

[46] Chang X, Xu T, Li Y, Wang K (2012). Dynamic modular architecture of protein-protein interaction networks beyond the dichotomy of ‘date’ and ‘party’ hubs. Nature.

[47] Bartomeus I, Ascher JS, Gibbs J, Danforth BN, Wagner DL, Hedtke SM (2013). Historical changes in northeastern US bee pollinators related to shared ecological traits. Proc Natl Acad Sci.

[48] Felsenstein J (1985). Phylogenies and the comparative method. Am Nat.

[49] Thompson JN (2005). The geographic mosaic of coevolution.

[50] Cadotte MW (2017). Functional traits explain ecosystem function through opposing mechanisms. Ecol Lett.

[51] Miliczky E (2008). Observations on the nesting biology of andrena (Plastandrena) prunorum Cockerell in Washington State (Hymenoptera: Andrenidae). J Kansas Entomol Soc.

[52] Evans MM. Effects of grazing and landscape on bee pollinators and their floral resources in rough fescue grasslands. University of Calgary (Master’s Thesis) 2013; p. 136.

[53] Dormann CF, Strauss R (2014). A method for detecting modules in quantitative bipartite networks. Methods Ecol Evol.

[54] Wagenmakers E, Farrell S (2004). AIC model selection using Akaike weights. Psychon Bull Rev.

[55] Burnham KP, Anderson DR (2001). Kullback–Leibler information as a basis ofr strong inference in ecological studies. Wildl Res.

[56] Blüthgen N, Fründ J, Vázquez DP, Menzel F (2015). What do interaction network metrics tell us about specialization and biological traits. Ecology.

[57] Almeida-Neto M, Guimaraes PRJ, Loyota RD, Ulrich W (2008). A consistent metric for nestedness analysis in ecological systems: reconciling concept and measurement. Oikos.

[58] Guimer R, Sales-Pardo M, Amaral AN (2008). Module identification in bipartite and directed networks. Phys Rev.

[59] Webb CO, Donoghue MJ (2005). Phylomatic: tree assembly for applied phylogenetics. Mol Ecol Notes.

[60] Wikstrom N, Savolainen V, Chase MW (2001). Evolution of the angiosperms: calibrating the family tree. Proc R Soc.

[61] Cadotte MW, Jonathan Davies T, Regetz J, Kembel SW, Cleland E, Oakley TH (2010). Phylogenetic diversity metrics for ecological communities: integrating species richness, abundance and evolutionary history. Ecol Lett.

[62] Münkemüller T, Lavergne S, Bzeznik B, Dray S, Jombart T, Schiffers K (2012). How to measure and test phylogenetic signal. Methods Ecol Evol.

[63] Parra JL, McGuire JA, Graham CH (2010). Incorporating clade identity in analyses of phylogenetic community structure: an example with hummingbirds. Am Nat.

[64] Blomberg SP, Garland T, Ives AR (2003). Testing for phylogenetic signal in comparative data: behavioral traits are more labile. Evolution.

[65] Kembel SW (2009). Disentangling niche and neutral influences on community assembly: assessing the performance of community phylogenetic structure tests. Ecol Lett.

