# Supplementary material for: Specialization in plant–pollinator networks: insights from local-scale interactions in Glenbow Ranch Provincial Park in Alberta, Canada
Source: BMC Ecol. 2019 Sep 6;19:34. doi: 10.1186/s12898-019-0250-z (PMC6731600; doi:10.1186/s12898-019-0250-z)
Supplement: Supplementary file 1 — Additional file 1: Appendix S1. Species traits and abundance data for plants and pollinators in Glenbow Ranch. [file 12898_2019_250_MOESM1_ESM.docx]

Appendix S1. Species traits and abundance data for plants and pollinators in Glenbow Ranch.

| site | Plant specie | d'plant | symmetry | flower abundance |
| --- | --- | --- | --- | --- |
| disturbed | *Achillea millefolium* | 0.7 | A | 4 |
| disturbed | *Allium cernuum* | 0.6 | Z | 15 |
| disturbed | *Anemone canadiensis* | 0.7 | A | 13 |
| disturbed | *Anemone cylindrica* | 1 | A | 5 |
| disturbed | *Aster falcata* | 0.5 | A | 18 |
| disturbed | *Campanula rotundifolia* | 0.7 | Z | 39 |
| disturbed | *Cirsium arvense* | 0.5 | A | 9 |
| disturbed | *Cirsium vulgare* | 0.6 | A | 2 |
| disturbed | *Erigeron caespitosus* | 0.6 | A | 1 |
| disturbed | *Erigeron glabellus* | 0.6 | A | 1 |
| disturbed | *Gaillardia aristata* | 0.2 | Z | 1 |
| disturbed | *Galium boreale* | 0.3 | Z | 1 |
| disturbed | *Geum aleppicum* | 0.7 | A | 1 |
| disturbed | *Monarda fistulosa* | 0.3 | Z | 9 |
| disturbed | *Potentilla fruticosa* | 0.4 | A | 64 |
| disturbed | *Potentilla hippiana* | 0.4 | A | 17 |
| disturbed | *Solidago missouriensis* | 0.3 | Z | 69 |
| disturbed | *Sonchus asper* | 0.3 | A | 2 |
| disturbed | *Symphoricarpos albus* | 0.6 | A | 71 |
| undisturbed | *Allium cernuum* | 0.5 | Z | 4 |
| undisturbed | *Aster falcata* | 0.8 | A | 2 |
| undisturbed | *Aster laevis* | 0.6 | A | 106 |
| undisturbed | *Campanula rotundifolia* | 0.5 | Z | 37 |
| undisturbed | *Cirsium arvense* | 0.3 | A | 8 |
| undisturbed | *Dalea purpurea* | 0.8 | Z | 13 |
| undisturbed | *Gaillardia aristata* | 0.7 | Z | 6 |
| undisturbed | *Hedysarum alpinum* | 0 | Z | 6 |
| undisturbed | *Hedysarum boreale* | 0.4 | A | 9 |
| undisturbed | *Heterotheca villosa* | 0.7 | Z | 2 |
| undisturbed | *Lathyrus ochroleucus* | 1 | Z | 3 |
| undisturbed | *Melilotus officinalis* | 0.1 | Z | 12 |
| undisturbed | *Monarda fistulosa* | 0.7 | Z | 1 |
| undisturbed | *Orthilia secunda* | 0.6 | Z | 1 |
| undisturbed | *Potentilla fruticosa* | 0.6 | A | 183 |
| undisturbed | *Rosa acicularis* | 0.7 | A | 9 |
| undisturbed | *Rubus pubescens* | 1 | A | 4 |
| undisturbed | *Senecio canus* | 1 | A | 1 |
| undisturbed | *Sisyrinchium montanum* | 0.4 | A | 1 |
| undisturbed | *Solidago missouriensis* | 0.5 | Z | 58 |
| undisturbed | *Symphoricarpos albus* | 0.6 | A | 16 |
| undisturbed | *Vicia americana* | 1 | Z | 8 |
| undisturbed | *Zigadenus elegans* | 0.7 | A | 11 |

| site | Pollinator species | d'poll | Size (mm) | Size categ | Sociality |
| --- | --- | --- | --- | --- | --- |
| disturbed | *Alydidae sp* | 0.4 | 14 | large | solitary |
| disturbed | *Anastoechus sp* | 0 | 11 | small | no |
| disturbed | *Andrena sp.y* | 0.5 | 12.5 | large | solitary |
| disturbed | *Anthocoridae sp* | 0 | 3 | small | no |
| disturbed | *Anthophora sp* | 0.6 | 11 | small | social |
| disturbed | *Apis mellifera* | 0.4 | 4 | small | social |
| disturbed | *Ashmeadiella cactorum* | 0.1 | 17.5 | large | solitary |
| disturbed | *Atactopsis sp1* | 0 | 15.3 | large | no |
| disturbed | *Bombus centralis* | 0.4 | 19 | large | social |
| disturbed | *Bombus fervidus* | 0.4 | 22 | large | social |
| disturbed | *Bombus rufocinctus* | 0.4 | 16 | large | social |
| disturbed | *Bombus sp* | 0 | 13 | large | social |
| disturbed | *Bombus ternarius* | 0.1 | 13 | large | social |
| disturbed | *Bombylius sp* | 0.2 | 10.5 | small | no |
| disturbed | *Caenotus sp* | 0.5 | 8 | small | no |
| disturbed | *Chrysomelidae sp* | 0.2 | 18 | large | no |
| disturbed | *Coelioxys sp* | 0 | 9.5 | small | no |
| disturbed | *Colletes sp* | 0 | 11 | small | solitary |
| disturbed | *Dolichovespula arenaria* | 0.4 | 12.5 | large | solitary |
| disturbed | *Dolichovespula norwegica* | 0.1 | 15 | large | solitary |
| disturbed | *Drymeia sp* | 0.1 | 4 | small | no |
| disturbed | *Dufourea sp* | 0.7 | 10 | small | social |
| disturbed | *Epistrophe sp* | 0 | 11 | small | no |
| disturbed | *Everes amyntula* | 0.8 | 25 | large | no |
| disturbed | *Gymnosoma sp* | 0 | 10 | small | no |
| disturbed | *Hesperapis sp* | 0.7 | 17.5 | large | no |
| disturbed | *Hydrotaea sp* | 0 | 6 | small | no |
| disturbed | *Hylaeus sp* | 0 | 14.6 | large | solitary |
| disturbed | *Ichneumonidae sp* | 0 | 12 | large | solitary |
| disturbed | *Lasioglossum sp* | 0.2 | 14.6 | large | social |
| disturbed | *Lispoides aequifrons* | 0 | 8.5 | small | no |
| disturbed | *Lycaeides melissa* | 0.4 | 14.5 | large | no |
| disturbed | *Megachile sp* | 0.1 | 15.3 | large | solitary |
| disturbed | *Merodon equestris* | 0.4 | 16 | large | no |
| disturbed | *Miridae sp* | 0.5 | 5 | small | no |
| disturbed | *Musca sp* | 0.4 | 8 | small | no |
| disturbed | *Nausigaster sp* | 0 | 8 | small | no |
| disturbed | *Neoascia globosa* | 0.4 | 5 | small | no |
| disturbed | *Noctuidae sp* | 0 | 25 | large | no |
| disturbed | *Onychogonia sp* | 0 | 11 | small | no |
| disturbed | *Paragus sp* | 0.6 | 4 | small | no |
| disturbed | *Parasyrphus sp* | 0.4 | 5 | small | no |
| disturbed | *Phyciodes cocyta* | 0 | 20 | large | no |
| disturbed | *Phyciodes pratensis* | 0.8 | 16 | large | no |
| disturbed | *Pipiza sp* | 0 | 9 | small | no |
| disturbed | *Pseudopanurgus sp* | 0.1 | 15.3 | large | solitary |
| disturbed | *Rhopalolemma sp* | 0.4 | 12 | large | solitary |
| disturbed | *Sericomyia sp* | 0.1 | 16 | large | no |
| disturbed | *Siphosturmia sp* | 0.7 | 15 | large | no |
| disturbed | *Spallanzania sp* | 0 | 50 | large | no |
| disturbed | *Speyeria cibele* | 0 | 15 | large | no |
| disturbed | *Speyeria sp* | 0.7 | 21.6 | large | no |
| disturbed | *Sphaerophoria sp* | 0.2 | 9.8 | small | no |
| disturbed | *Tenthredinidae sp* | 0 | 20 | large | solitary |
| disturbed | *Thymelicus lineola* | 0.5 | 15.4 | large | no |
| disturbed | *Trichopsomyia sp* | 0 | 4 | small | no |
| disturbed | *Vespula acadica* | 0 | 15 | large | social |
| disturbed | *Vespula consobrina* | 0.4 | 12 | large | social |
| disturbed | *Vespula pensylvanica* | 0.4 | 13 | large | social |
| disturbed | *Xanthogramma sp* | 0.4 | 9 | small | no |
| undisturbed | *Andrena sp* | 0.1 | 12.5 | large | solitary |
| undisturbed | *Anthomyiidae sp* | 0 | 8 | small | no |
| undisturbed | *Anthophora sp* | 0 | 11 | small | social |
| undisturbed | *Apis mellifera* | 0.9 | 4 | small | social |
| undisturbed | *Ashmeadiella cactorum* | 0 | 17.5 | large | solitary |
| undisturbed | *Bombus bifarius* | 0 | 12 | large | social |
| undisturbed | *Bombus californicus* | 0.7 | 14 | large | social |
| undisturbed | *Bombus centralis* | 0.7 | 19 | large | social |
| undisturbed | *Bombus frigidus* | 0.5 | 17.5 | large | social |
| undisturbed | *Bombus huntii* | 0.1 | 16 | large | social |
| undisturbed | *Bombus insularis* | 0.7 | 14.5 | large | social |
| undisturbed | *Bombus melanopygus* | 0.5 | 14 | large | social |
| undisturbed | *Bombus nevadensis* | 0.8 | 15.5 | large | social |
| undisturbed | *Bombus rufocinctus* | 0.2 | 16 | large | social |
| undisturbed | *Bombus sitkensis* | 0 | 15 | large | social |
| undisturbed | *Bombus sp* | 0.5 | 13 | large | social |
| undisturbed | *Bombus ternarius* | 0.1 | 13 | large | social |
| undisturbed | *Bombus vagans* | 0.3 | 12 | large | social |
| undisturbed | *Buquetia sp* | 0.2 | 3 | small | no |
| undisturbed | *Caenotus sp* | 0 | 8 | small | no |
| undisturbed | *Celastrina ladon* | 1 | 20 | large | no |
| undisturbed | *Cercyonis pegala* | 0.7 | 30 | large | no |
| undisturbed | *Chaetogaedia sp* | 0 | 10 | small | no |
| undisturbed | *Chrysomelidae sp* | 0.5 | 18 | large | no |
| undisturbed | *Chrysotoxum sp* | 0.1 | 14.5 | large | no |
| undisturbed | *Cleridae sp1* | 0.5 | 20.5 | large | no |
| undisturbed | *Colias philodice* | 0.2 | 22 | large | no |
| undisturbed | *Drymeia sp* | 0.3 | 4 | small | no |
| undisturbed | *Dufourea sp* | 0.6 | 10 | small | social |
| undisturbed | *Elampus hyalinus* | 0 | 10 | small | no |
| undisturbed | *Epistrophe sp* | 0 | 11 | small | no |
| undisturbed | *Eremomyia sp* | 0 | 10.5 | small | no |
| undisturbed | *Eumenes crucifera* | 0 | 20 | large | no |
| undisturbed | *Euphilotes ancilla* | 0.8 | 18.5 | large | no |
| undisturbed | *Halictus sp* | 0.1 | 10.5 | small | social |
| undisturbed | *Helophilus sp* | 0.5 | 13 | large | no |
| undisturbed | *Hesperapis sp* | 0.2 | 17.5 | large | no |
| undisturbed | *Hesperia sp* | 0.1 | 15 | large | no |
| undisturbed | *Hydrotaea sp* | 0.1 | 6 | small | no |
| undisturbed | *Hylaeus sp* | 0.2 | 14.6 | large | solitary |
| undisturbed | *Lasioglossum sp* | 0.6 | 14.6 | large | social |
| undisturbed | *Limnophora sp* | 0 | 6 | small | no |
| undisturbed | *Lycaenidae sp* | 0 | 45 | large | no |
| undisturbed | *Lydina sp* | 0.1 | 7 | small | no |
| undisturbed | *Masistylum sp* | 0 | 12 | large | no |
| undisturbed | *Megachile sp* | 0.2 | 15.3 | large | solitary |
| undisturbed | *Melangyna triangulifera* | 0 | 8 | small | no |
| undisturbed | *Merodon equestris* | 0 | 13 | large | no |
| undisturbed | *Miridae sp* | 0.7 | 5 | small | no |
| undisturbed | *Musca sp* | 0.2 | 8 | small | no |
| undisturbed | *Paragus sp* | 0 | 4 | small | no |
| undisturbed | *Phyciodes cocyta* | 1 | 16 | large | no |
| undisturbed | *Pompilidae sp* | 0.7 | 15 | large | no |
| undisturbed | *Potamia sp* | 0 | 12 | large | no |
| undisturbed | *Pseudochirosia sp* | 0 | 7 | small | no |
| undisturbed | *Pseudopanurgus sp* | 0 | 15.3 | large | solitary |
| undisturbed | *Rhopalolemma sp* | 0 | 12 | large | solitary |
| undisturbed | *Siphosturmia sp* | 0.4 | 15 | large | no |
| undisturbed | *Spallanzania sp* | 0.4 | 50 | large | no |
| undisturbed | *Speyeria hesperis* | 0.7 | 9.8 | small | no |
| undisturbed | *Sphaerophoria sp* | 0.1 | 13 | large | no |
| undisturbed | *Spilomyia sp* | 0.1 | 10 | small | no |
| undisturbed | *Syrphus sp* | 0.1 | 20 | large | no |
| undisturbed | *Tenthredinidae sp* | 0 | 5 | small | solitary |
| undisturbed | *Thricops sp* | 0.3 | 5 | small | no |
| undisturbed | *Thymelicus lineola* | 0.5 | 15.4 | large | no |
| undisturbed | *Villa sp* | 0.4 | 11 | small | no |
| undisturbed | *Volucella sp* | 0 | 15 | large | no |
| undisturbed | *Xanthogramma sp* | 0.3 | 9 | small | no |

*‘no’= species that did not qualify for the categorization of exhibiting sociality.
