# Supplementary figures and images for: Specialization in plant–pollinator networks: insights from local-scale interactions in Glenbow Ranch Provincial Park in Alberta, Canada
Source: BMC Ecol. 2019 Sep 6;19:34. doi: 10.1186/s12898-019-0250-z (PMC6731600; doi:10.1186/s12898-019-0250-z)

**A. Bray-Curtis plant dissimilarity**

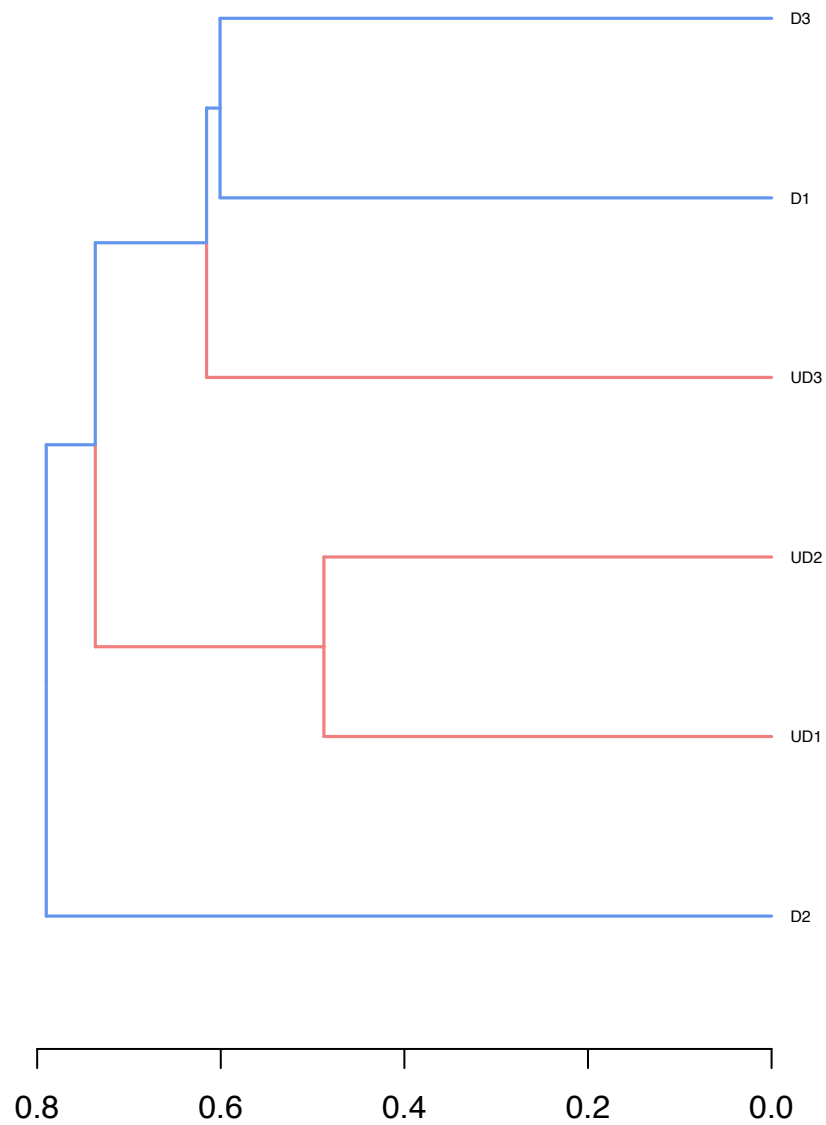

**B. Bray-Curtis pollinator dissimilarity**

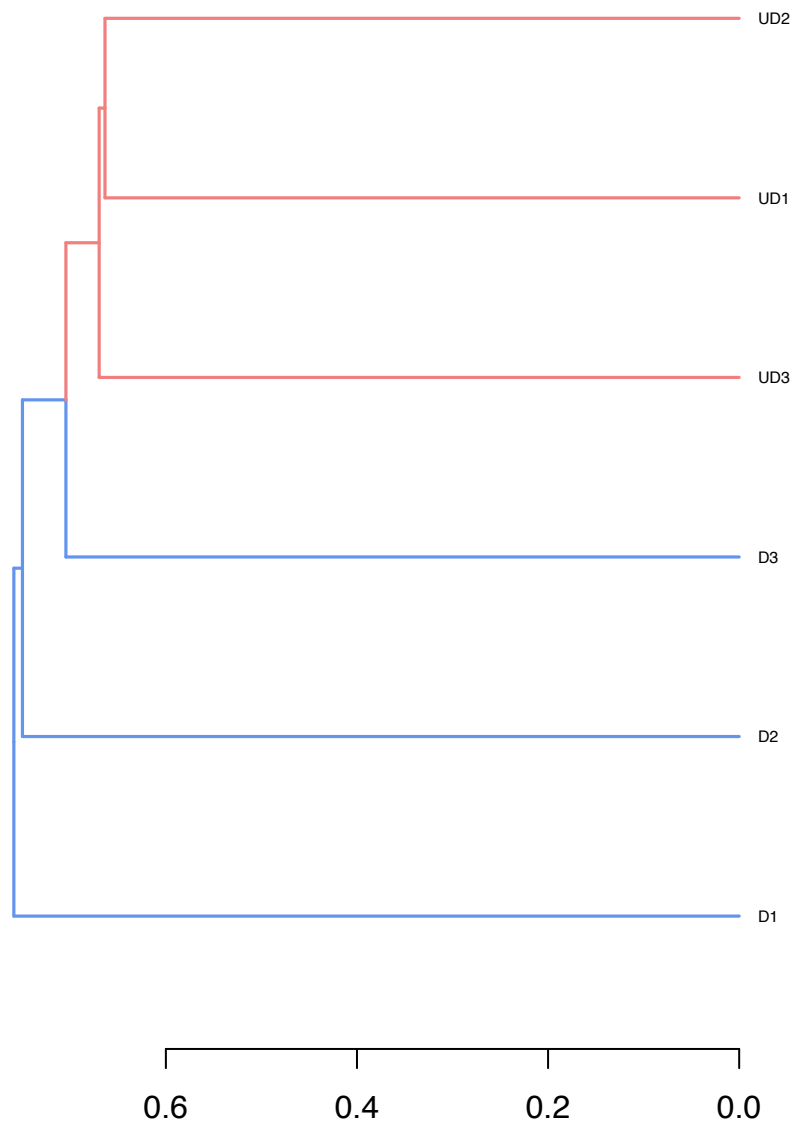

Supplement: Supplementary file 2 — Additional file 2: Figure S1. Bray-Curtis dissimilarity index of visitor and plant composition of undisturbed (UD) and disturbed (D) sites. The index approaches 0 when samples are similar and approaches 1 when assemblages are different. Visitors composition differ in their clade compositional structure between undisturbed and disturbed sites. [file 12898_2019_250_MOESM2_ESM.pdf]
